# Interrupting Prolonged Sitting with Regular Activity Breaks does not Acutely Influence Appetite: A Randomised Controlled Trial

**DOI:** 10.3390/nu10020125

**Published:** 2018-01-26

**Authors:** Evelyn M. Mete, Tracy L. Perry, Jillian J. Haszard, Ashleigh R. Homer, Stephen P. Fenemor, Nancy J. Rehrer, C. Murray Skeaff, Meredith C. Peddie

**Affiliations:** 1 Department of Human Nutrition, University of Otago, Dunedin 9016, New Zealand; evelyn.mete@postgrad.otago.ac.nz (E.M.M.); tracy.perry@otago.ac.nz (T.L.P.); jill.haszard@otago.ac.nz (J.J.H.); ash.homer@postgrad.otago.ac.nz (A.R.H.); murray.skeaff@otago.ac.nz (C.M.S.); 2 School of Physical Education, Sport, and Exercise Sciences, University of Otago, Dunedin 9016, New Zealand; stephen.fenemor@otago.ac.nz (S.P.F.); nancy.rehrer@otago.ac.nz (N.J.R.)

**Keywords:** regular activity breaks, sedentary, appetite, randomized controlled trial

## Abstract

Regular activity breaks increase energy expenditure; however, this may promote compensatory eating behaviour. The present study compared the effects of regular activity breaks and prolonged sitting on appetite. In a randomised, cross-over trial, 36 healthy adults (BMI (Body Mass Index) 23.9 kg/m^2^ (S.D. = 3.9)) completed four, two-day interventions: two with prolonged sitting (SIT), and two with sitting and 2 min of walking every 30 min (RAB). Standardized meals were provided throughout the intervention, with an *ad libitum* meal at the end of Day 2. Appetite and satiety were assessed throughout both days of each intervention using five visual analogue scales. The five responses were combined into a single appetite response at each time point. The area under the appetite response curve (AUC) was calculated for each day. Intervention effects for appetite response AUC and *ad libitum* meal intake were tested using linear mixed models. Appetite AUC did not differ between interventions (standardised effect of RAB compared to SIT: Day 1: 0.11; 95% CI: −0.28, 0.06; *p* = 0.212; Day 2: 0.04; 95% CI: −0.15, 0.24; *p* = 0.648). There was no significant difference in energy consumed at the *ad libitum* lunch meal on Day 2 between RAB and SIT. Interrupting prolonged sitting with regular activity breaks does not acutely influence appetite or volume of food consumed, despite inferred increases in energy expenditure. Longer-term investigation into the effects of regular activity breaks on energy balance is warranted.

## 1. Introduction

Sedentary behaviour, defined as activities of low energy expenditure performed in a seated or reclining posture [1], is a risk factor for detrimental health outcomes such as type 2 diabetes, cardiovascular disease, and all-cause mortality [2,3]. Regularly interrupting (every 20–60 min) sedentary time with short (1 min 40 s to 5 min) bouts of walking acutely improves postprandial plasma glucose and insulin responses [4,5,6,7].

Performing activity breaks during sedentary behaviours increases energy expenditure [8,9], which may contribute to a meaningful negative energy balance if not accompanied by increased energy intake. Conversely, engaging in extended bouts of sedentary time reduces the opportunity for energy expenditure and poses challenges to achieving energy balance [10]. It is uncertain if and to what extent compensatory changes in energy intake and expenditure occur in response to intentional energy expenditure [11]. If appetite were to increase in response to short bouts of intentional energy expenditure, energy intake may also increase; however, if appetite remains unaffected, energy intake may remain unchanged despite the increased energy expenditure, resulting in a change in energy balance.

Experimental evidence from studies designed to measure the acute effect of regular activity breaks on appetite response is scarce, but the results seem to indicate that walking for 2 min at a light intensity every 30 min, or for 5 min at a moderate intensity every 60 min, does not increase appetite [12,13,14]. However, to date, all of the studies in adults have measured appetite response over a single 6–12 h session of activity breaks, and only one [12] has investigated whether possible changes in appetite are reflected in changes in the amount of food consumed. Therefore, the primary aim of this study was to determine the effect on appetite when prolonged sitting is interrupted by regular activity breaks over a 2-day intervention period. However, changes in appetite are only meaningful if they result in changes in energy intake; therefore, the secondary aim was to determine whether appetite predicted energy intake at an *ad libitum* meal.

## 2. Materials and Methods

### 2.1. Study Design

This randomised, controlled, cross-over intervention trial was conducted at the University of Otago, New Zealand between June 2014 and November 2016. This paper presents secondary analysis from the primary study, which measured changes in blood glucose and triglycerides, and provides a detailed description of methodology [15]. The University of Otago Human Ethics committee approved the study (number 13/112) in April 2013. This trial is registered with the Australian New Zealand Clinical Trials Registry as ANZCTR12614000624684.

### 2.2. Participants

Eligible participants (*n* = 36) were English-speaking, aged 18–40 years old, not pregnant or planning pregnancy within the next four months, non-smoking, and without allergies or intolerances to gluten or dairy. They had no history of diabetes, cardiovascular disease, or other chronic disease affecting carbohydrate or lipid metabolism. They were engaged in sedentary occupations and did not habitually meet current physical activity guidelines, i.e., they completed less than 150 min of moderate-to-vigorous physical activity each week.

Participants were excluded if they exhibited any of the following: fasting blood glucose > 6.1 mmol/L; systolic or diastolic blood pressure above 140 or 90 mmHg, respectively; fasting total cholesterol concentration > 6 mmol/L, fasting triglyceride concentration > 2.5 mmol/L.

All subjects gave their informed consent for inclusion before they participated in the study.

### 2.3. Preliminary Testing

All participants attended a screening visit in which they provided a fasting blood sample to measure blood glucose and lipid concentrations. Measures of blood pressure and anthropometry were taken. Further details about the preliminary testing can be found here: [15].

At least seven days prior to their initial intervention session, participants completed a maximal aerobic capacity assessment to determine the individualised speed and incline that would elicit 60% maximal aerobic capacity [15]. This intensity was chosen, as it has previously been shown to result in changes in postprandial glucose and insulin responses in young healthy individuals [7].

### 2.4. Experimental Protocol

**Figure 1:**
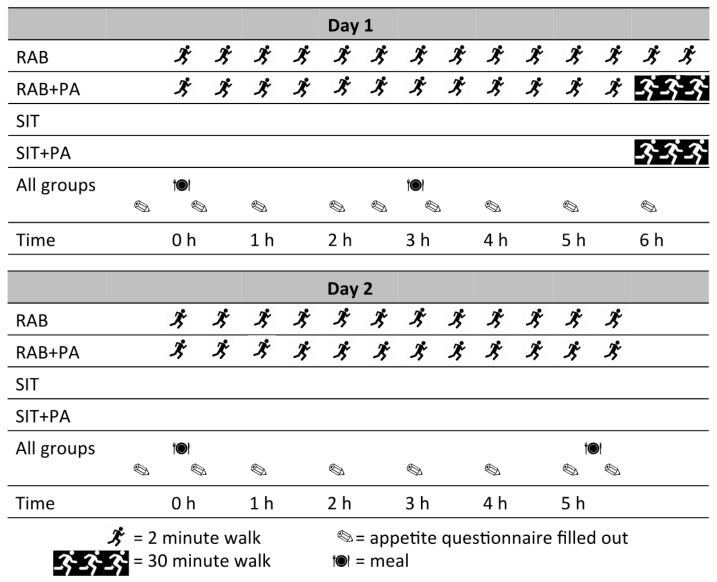
Timing of activities, meals, and questionnaires on Days 1 and 2 of each intervention. RAB: regular activity breaks; RAB+PA: regular activity breaks with physical activity; SIT: prolonged sitting; SIT+PA: prolonged sitting with physical activity.

Participants completed four two-day interventions, each separated by a washout of at least five days. Time spent at the research clinic was 6.5 h on Day 1 and 5 h on Day 2, during which times an investigator was present in the room with participants to ensure compliance with all protocols. The interventions are shown in Figure 1 and described as follows:

(1) Prolonged Sitting (SIT): for both days, participants remained continuously seated, only moving from their chair to visit the bathroom when required (located six metres from seating area).
(2) Prolonged Sitting with Physical Activity (SIT+PA^D1^): for both days, participants remained continuously seated, except for completing a 30 min treadmill walk at a speed and incline to elicit 60% VO_2max_ at the end of Day 1 only. Time spent physically active: 30 min.
(3) Regular Activity Breaks (RAB): for both days, sitting was interrupted every 28 min with a 2 min bout of walking on the treadmill at a speed and incline to elicit 60% VO_2max_ Time spent physically active: 48 min.
(4) Regular Activity Breaks with Physical Activity (RAB+PA^D1^): for both days, sitting was interrupted every 28 min with a 2 min bout of walking on the treadmill at a speed and incline to elicit 60% VO_2max_. In addition, at the end of Day 1 only participants completed a 30 min bout of continuous walking on the treadmill at a speed an incline to elicit 60% VO_2max_. Time spent physically active: 74 min.

The timing of the physical activity at the end of Day 1 meant it was impossible to affect appetite scores on Day 1 and unlikely to influence appetite scores on Day 2. Hence, the SIT and SIT+PA^D1^ interventions were considered as a single, repeated intervention (“SIT^combine^”), and the RAB and RAB+PA^D1^ were similarly considered as a single, repeated intervention (“RAB^combine^”).

### 2.5. Randomisation

Stata software (version 11.2 for MAC; StataCorp, College Station, TX, USA) was used to randomly assign participants to complete the four interventions in one of 12 possible orders, using a Williams design [16]. The randomization sequence was generated prior to recruitment and concealed electronically.

### 2.6. Standardisation

Participants avoided moderate to vigorous physical activity for three days preceding each intervention, and abstained from alcohol consumption for the preceding 24 h. All participants provided verbal confirmation of adherence to these protocols. During the 24 h prior to their first intervention, participants recorded all foods and drinks, including tea and coffee consumed, and replicated this regime before all subsequent interventions. Between Day 1 and Day 2 of each intervention, participants were asked to keep physical activity to a minimum.

### 2.7. Meal Composition

Energy and macronutrient composition of all standardised meals (Table 1) provided to participants were calculated using nutrition information provided by the food manufacturers where possible and the New Zealand Food Composition Tables [17].

The meals provided on Day 1 were designed to adhere to the Ministry of Health macronutrient guidelines for percentage total energy contributed by each nutrient [18]. Energy intake on Day 1 for each participant was calculated using the average reported energy intake in the August 2007 New Zealand Adult Nutrition Survey [19]; this was then converted to kJ/kg. Participants received breakfast and lunch at the study site on Day 1 at approximately 0900 h and 1200 h, respectively. Breakfast consisted of muesli, milk, and canned peaches, and lunch consisted of pumpkin soup, a bread roll with margarine, and an apple. Participants were given a dinner to take home and consume between 1900 h and 2000 h. This consisted of lasagne, broccoli, carrots, yoghurt, and orange pieces. The breakfast meal on Day 2 was the meal for which postprandial response was measured in the primary study, and as such was designed to provide 0.7 g/kg of fat and consisted of croissants and cheese with margarine.

Throughout each participant’s first intervention, water was consumed *ad libitum*, and then matched on subsequent interventions.

### 2.8. Ad Libitum Meal Protocol

An *ad libitum* meal was offered at the end of Day 2 at approximately 1300 h and approximately 10–15 min after the last 2 min walk. This consisted of three packets of *Wattie’s Steam Fresh Meal Sensation—Thai Chicken Noodle*, which amounted to approximately 900 g. This meal provided 4650 kJ in total and contained 515 kJ/100 g energy, 13.8 g/100 g carbohydrate, 3.9 g/100 g fat, and 7.2 g/100 g protein. In order to calculate the exact amount consumed, each meal was weighed before being served, and the remainder was weighed at the end of the meal. The meal was provided in a large bowl, from which participants served themselves. Participants were instructed to eat until comfortably full.

### 2.9. Assessment of Appetite Response

At baseline, immediately prior to and following meals, and hourly throughout both days of each intervention session, participants completed an appetite questionnaire which covered five aspects of appetite: hunger (“How hungry do you feel right now?”), satisfaction (“How satisfied do you feel?”), fullness (“How full do you feel?”), quantity of food (“How much do you think you can eat?”), and desire for food (“Would you like to eat something right now?”). Text was anchored at each end of the 100 mm visual analogue scales (“Not at all/not much”, “Very much/a lot”) and participants were asked to mark the scale in accordance with how they felt. The appetite questionnaire (available on request from the authors) was adapted from Flint et al. [20], based on methodology used by Farah & Gill [21]. An overall appetite score for each participant at each time point was calculated from the mean of all five appetite responses. Cronbach’s α values for the appetite scale at each time point ranged from 0.81 to 0.96, with an overall mean Cronbach’s α of 0.91, indicating uni-dimensionality among the five appetite questions, confirming the appropriateness of creating a single appetite score.

The area under the appetite score response curve (AUC) was calculated using the Stata command “pkexamine”, using cubic splines, for the three hours following breakfast and for the whole day, for Day 1 and Day 2 separately. The days were treated separately because the breakfasts and the timing of the day differed substantially. The area under the curve was only calculated if the participant was not missing appetite scores at the beginning or end of the measurement period. From 2448 administrations of the appetite questionnaire, AUC could not be calculated on three occasions (two were missing the last appetite measure for the day; one was missing more than three appetite scores for the day). However, as a result of the combination of the RAB and RAB+PA^D1^ and SIT and SIT+PA^D1^ conditions, all participants with plausible responses had at least one AUC calculated from the two combined conditions, and therefore no participants were excluded on the basis of missing data.

To confirm that there was not an effect of the physical activity intervention on Day 2’s appetite response, a two-tailed, paired t-test compared RAB to RAB+PA^D1^ and SIT to SIT+PA^D1^. No meaningful differences were found for any of the appetite measures (all *p* > 0.05).

### 2.10. Statistical Analysis

Stata software (Version 14.2 for PC; StataCorp, College Station, TX, USA) was used for all statistical analysis.

Mixed model regression was used to determine differences in appetite AUC, appetite scores prior to *ad libitum* lunch, change in appetite scores before and after lunch, and volume of food consumed between SIT^combine^ and RAB^combine^ interventions, adjusted for period (the number of the intervention session for that participant) and randomised order, with participant ID as a random effect. The study was powered to detect a clinically meaningful change in postprandial glucose AUC; therefore, to aid in interpretation of differences in appetite response, standardized differences and 95% confidence intervals were also calculated and presented. Overall mean appetite scores for the whole sample at each time for both conditions were plotted for each day to illustrate the average appetite trajectories.

To investigate whether appetite score predicted energy intake during an *ad libitum* lunch, irrespective of intervention, mixed model regression was also used, with the amount consumed as the outcome variable and the appetite score immediately preceding lunch as the predictor variable, adjusted for intervention, period, and order, with participant ID as a random effect. Assumptions of the models were checked and found to be met in all cases.

## 3. Results

**Figure 2:**
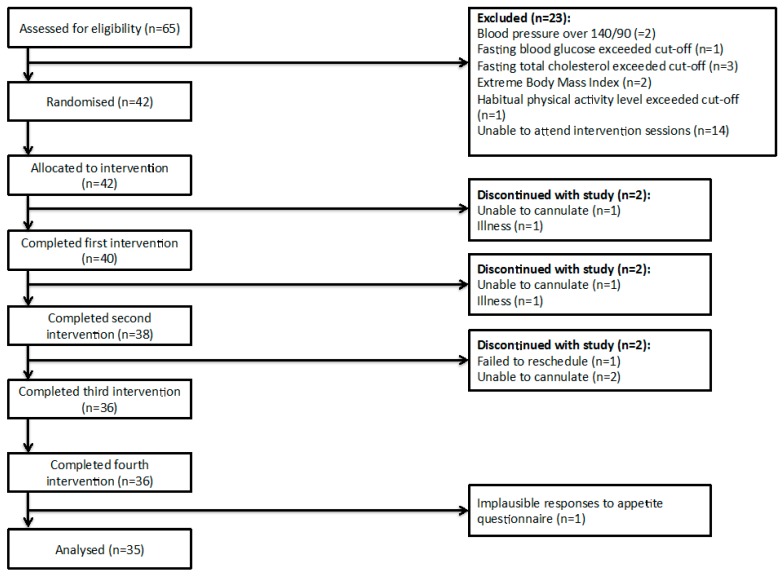
Consort diagram.

Of sixty-five participants screened, forty-two met inclusion and exclusion criteria and were allocated to an intervention order. During the study, six participants dropped out due to illness or being unable to cannulate. Thirty-six participants completed all four interventions. Prior to analysis, one more participant was excluded due to implausible appetite scores. Therefore, 35 participants were included in analysis (Figure 2). Participant characteristics and cardiovascular health biomarkers at the time of recruitment are shown in Table 2.

**Figure 3:**
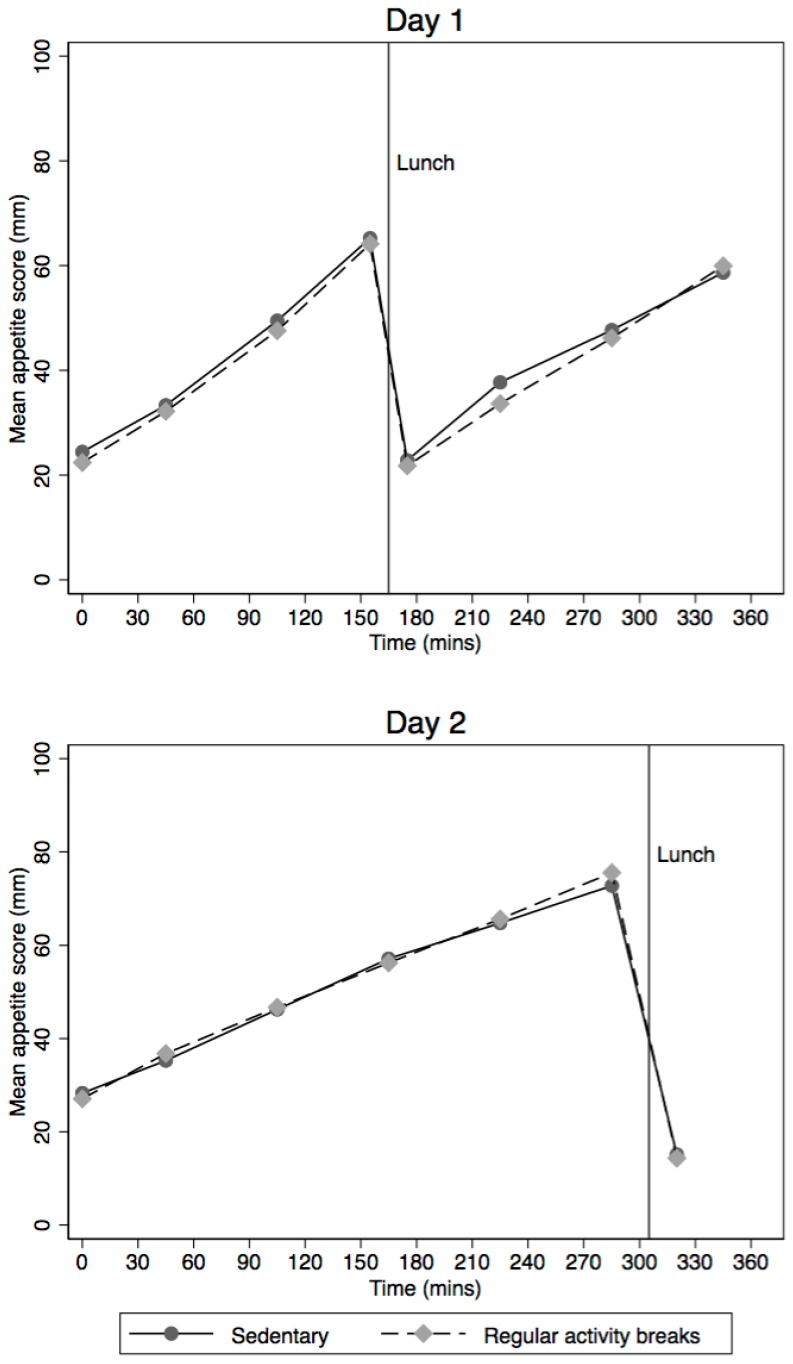
Average appetite scores for Days 1 and 2 of each intervention.

Average appetite scores across each day and for each intervention (SIT^combine^ and RAB^combine^) are shown in Figure 3. Regular activity breaks did not significantly change appetite response compared to prolonged sitting (Table 3). For Day 1, the standardised AUC difference between interventions was 0.11 S.D. (95% CI: −0.28, 0.06) and for Day 2 it was 0.04 S.D. (95% CI: −0.15, 0.24).

Quantity of food consumed at the *ad libitum* meal at the end of Day 2 did not differ significantly between RAB^combine^ and SIT^combine^ (mean difference 17 g; 95%CI: −12, 46; *p* = 0.253) (Table 4). The mean appetite score prior to the *ad libitum* lunch, and the mean change in appetite scores before and after the *ad libitum* lunch, did not significantly differ between interventions.

The appetite score before the *ad libitum* lunch significantly predicted the amount (in grams) consumed (β = −2.4 mm; 95% CI: 0.8 mm, 3.9 mm, *p* = 0.003). The mean appetite score before the *ad libitum* lunch was 74.3 mm (95% CI: 71.9, 76.8), and the mean appetite score following the *ad libitum* lunch was 15.1 mm (95% CI: 12.9, 17.2), in which 0 mm was the lowest score possible and 100 mm was the highest. The mean change in appetite score (adjusted for period, order, and intervention) was −59.3 mm (95% CI: −62.3, −56.4), *p* < 0.001.

## 4. Discussion

Performing regular activity breaks—2 min of brisk walking every 30 min—did not affect appetite response when compared to prolonged sitting, nor did it affect *ad libitum* intake of a meal. This is clearly demonstrated by the standardised effect sizes comparing appetite response between regular activity breaks and prolonged sitting, the magnitude of which does not approach a clinically meaningful effect (>0.5 S.D.) [22]. Evidently, performing regular activity breaks does not result in acute changes in either subjective measures of appetite or the objective measure of the amount of food, and thus energy, consumed afterwards. Results from previous studies have also indicated that performing regular activity breaks over a period of 5–12 h has little effect on appetite and *ad libitum* intake of a meal [12,13,14]. The results of the current study add to the small body of literature by extending the time frame over which appetite response is measured. Even after a period of ~30 h of strict control of energy intake and activity pattern, neither appetite nor *ad libitum* intake of a meal are affected by performing regular activity breaks.

Regular activity breaks have been estimated to increase energy intake by 100–670 kJ over an 8 h period [8,9]. Additionally, Bailey et al. estimated that regular activity breaks elicit a relative energy intake that is ~600–1400 kJ smaller than the energy intake when sitting, measured over a period of 5 h [12]. Although not reported here, participants in the current study expended approximately 1900 kJ more during the regular activity break intervention than during the prolonged sitting intervention [23]. Clearly, performing regular activity breaks has the potential to influence energy balance, as excess energy expenditure is not matched by either an increase in desire to eat, or actual dietary or energy intake. However, the implications of these results are not aligned with findings from a recently published meta-analysis of prospective observational studies, which indicate that sedentary time is not meaningfully associated with obesity or changes in body weight [24]. There are two possible explanations for this disparity. The first is that the negative energy balance established at the onset of regular activity breaks is compensated for over a period of weeks or months, either in terms of dietary intake or activity patterns, thus negating the changes we, and others, have observed. The second explanation is that the pattern in which sedentary time is accumulated may affect energy balance differently than total sedentary time. This idea is supported by cross-sectional analyses, which indicated that a greater number of breaks from sedentary time were associated with a smaller waist circumference, independent of total sedentary time and time spent in moderate to vigorous physical activity [25,26]. Furthermore, changes in waist circumference may represent changes in body composition, irrespective of bodyweight.

The use of both an *ad libitum* meal and visual analogue scales to measure appetite is a prominent strength of this study. Appetite scores just prior to the *ad libitum* meal significantly predicted the quantity consumed during this meal, providing further support for the use of the visual analogue scales to accurately describe appetite. Further strengths of this study were its strict standardisation procedures and randomised cross-over design. The major limitation of this study is that aspects of the study design, such as the timing of activity bouts in relation to meals, and the differing meal compositions used on Day 1 and Day 2, were driven by the primary outcomes of the study, and thus were perhaps not the ideal design for investigating the effects of regular activity breaks on appetite. However, we confirmed statistically that the continuous 30 min bout of activity performed at the end of Day 1 did not affect appetite responses on Day 2. Additionally, the different meal compositions used on Day 1 and Day 2 broaden the applicability of the results of this study, as they clearly illustrate that regular activity breaks do not influence appetite response regardless of whether the meal fed is a high carbohydrate or high fat meal.

Limitations include the fact that information on sleep prior to and between intervention days was not recorded. Sleep is known to effect glucose and appetite regulation [27], and regular activity breaks appear not to reduce glucose responses when sleep is restricted [28]. However, given the randomized crossover nature of this study, it seems unlikely that differences in sleep between interventions would have had a large effect on the results. A further potential limitation of our study was that it was powered to detect differences in postprandial glucose AUC rather than appetite. For this reason, standardised differences with confidence intervals were calculated to aid in comparison between interventions. Given these results, it is unlikely that a meaningful effect of regular activity breaks on appetite exists.

This is the largest randomised controlled cross-over trial to undertake a detailed investigation into the effect of regular activity breaks on appetite, when compared to prolonged sitting, and is also the first to do so over a two-day period. Both appetite and *ad libitum* food intake were unaffected by regular activity breaks compared to prolonged sitting, but were accompanied by a difference in energy expenditure of approximately 1900 kJ across two days. If appetite affects the amount we eat and regular activity breaks increase energy expenditure without an effect on appetite then the long-term influence of regular activity breaks on weight control could be important. Longer-term randomised controlled trials are required to ascertain whether this extra energy expenditure is compensated for over a longer period of time.

## Figures and Tables

**Table 1:** Energy and macronutrient composition of test meals on Days 1 and 2 of each intervention.

	Day One				Day Two
	Breakfast	Lunch	Dinner	Total	Breakfast
Energy (kJ/kg)	32.65	26.17	62.75	121.57	43.24
Carbohydrate (g/kg)	1.07	0.99	1.48	3.54	0.71
Fat (g/kg)	0.23	0.12	0.71	1.06	0.70
Protein (g/kg)	0.27	0.11	1.47	1.85	0.32

**Table 2:** Participant baseline characteristics.

Characteristic	Mean (S.D.)
Age (years)	25.4 (3.9)
Sex (female)	24 (69) ^a^
BMI (kg/m^2^)	23.9 (3.9)
Systolic blood pressure (mmHg)	121 (9)
Diastolic blood pressure (mmHg)	74 (8)
Minutes of weekly activity	120 (70)
Total cholesterol (mmol/L) ^b^	4.4 (0.7)
TAGs (mmol/L) ^b^	0.98 (0.37)
LDL cholesterol (mmol/L) ^b^	2.5 (0.68)
HDL cholesterol (mmol/L) ^b^	1.5 (0.35)
Blood glucose (mmol/L) ^b^	4.96 (0.37)

^a^ *n* (%); ^b^ Measured from a fasted blood sample.

**Table 3:** Difference in postprandial appetite response between regular activity breaks (RAB^combine^) and sedentary (SIT^combine^) interventions (*n* = 35).

	SIT^combine^ AUC Mean (S.D.) (mm·min)	RAB^combine^ AUC Mean (S.D.) (mm·min)	Mean Difference (95% CI) (mm·min)	Standardised Effect (95% CI)	*p*-Value ^a^
Appetite response to breakfast on Day 1	6624 (2427)	6403 (2297)	−225 (−730, 281)	−0.08 (−0.27, 0.11)	0.383
Appetite response to breakfast on Day 2	6882 (2522)	7049 (2379)	129 (−343, 601)	0.05 (−0.13, 0.23)	0.593
Combined ^b^ appetite response to breakfast	6783 (2330)	6726 (2164)	−55 (−437, 326)	−0.02 (−0.16, 0.12)	0.777
Overall ^c^ appetite response over Day 1	14,778 (5201)	14,191 (4750)	−583 (−1499, 333)	0.11 (−0.28, 0.06)	0.212
Overall ^c^ appetite response over Day 2	16,800 (4058)	16,978 (3887)	195 (−643, 1033)	0.04 (−0.15, 0.24)	0.648

^a^ Adjusted for period and order, and also for day when comparing combined appetite response to breakfast; ^b^ appetite response to breakfast on day 1 and day 2 combined; ^c^ overall appetite response on day 1 was for 6 h with lunch at midday; overall appetite response on day 2 was for 5.5 h, not including lunch.

**Table 4:** Difference in *ad libitum* lunch consumption and appetite response to *ad libitum* lunch between regular activity breaks and sedentary interventions (*n* = 35).

	SIT^combine^ Mean (S.D.)	RAB^combine^ Mean (S.D.)	Mean Difference between Interventions (95% CI)	*p*-Value ^a^
Amount *ad libitum* lunch consumed (g)	470 (181)	487 (195)	17 (−12, 46)	0.253
Mean appetite score ^b^ before *ad libitum* lunch (mm)	73 (13)	76 (12)	2.6 (−6.0, 0.8)	0.131
Mean difference in appetite score ^b^ after *ad libitum* lunch compared to before (mm)	−58 (20)	−61 (17)	−0.8 (−3.7, 2.1)	0.594

^a^ Adjusted for period and order; for difference in appetite score after lunch also adjusted for appetite score before lunch; ^b^ score found by combining five questions on appetite that were measured on a 100 mm visual analogue scale giving a score between 0 and 100 mm.
